# Positive surgical margins in nephron-sparing surgery: risk factors and therapeutic consequences

**DOI:** 10.1186/1477-7819-12-252

**Published:** 2014-08-08

**Authors:** Julie Steinestel, Sandra Steffens, Konrad Steinestel, Andres Jan Schrader

**Affiliations:** Department of Urology, Münster University Medical Center, Albert-Schweitzer-Campus 1, Building A1, 48149 Münster, Germany; Department of Urology, Hannover University Medical School, Carl-Neubergstr. 1, 30625 Hannover, Germany; Department of Pathology, Bundeswehrkrankenhaus Ulm, Oberer Eselsberg 40, 89081 Ulm, Germany

**Keywords:** Renal cell carcinoma, Positive surgical margins, Therapeutic relevance, R1, Prognosis, Survival review

## Abstract

The increased use of nephron-sparing surgery to treat localized renal cell carcinoma (RCC) lends weight to the question of the value of microscopically positive surgical margins (PSM) in cases with a tumor bed macroscopically free of residual tumor. The aim of this article is to highlight the data available on risk factors for PSM, their clinical relevance, and possible therapeutic consequences. For this purpose, publications on the incidence and relevance of PSM after partial nephrectomy from the last 15 years were examined and evaluated. We summarize that PSM are generally rare, regardless of the surgical procedure, and are seen more often in connection with an imperative indication for nephron-sparing surgery as well as a central tumor location. Most studies describe that PSM lead to a moderate increase in the rate of local relapses, but no study has thus far been able to demonstrate an association with shorter tumor-specific overall survival. Intraoperative frozen section analysis had no positive influence on the risk of definite PSM in most trials. Therefore, we conclude that PSM should definitely be avoided. However, in cases with a macroscopically tumor-free intraoperative resection bed, they should lead to close surveillance of the affected kidney and not to immediate (re)intervention.

## Introduction

**Figure 1:**
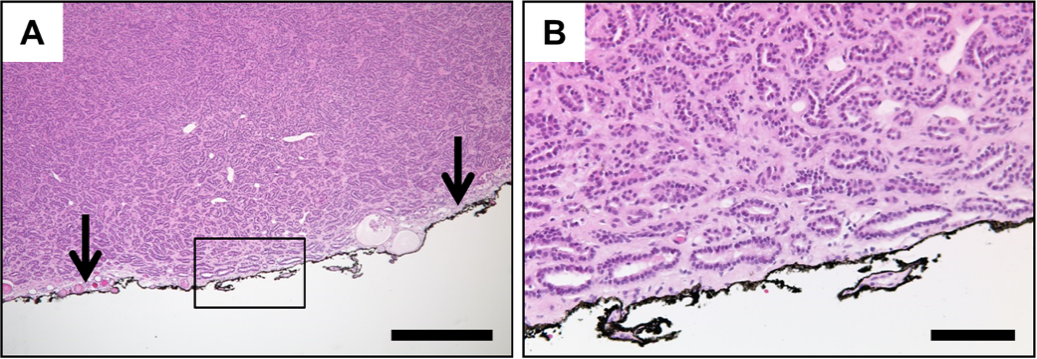
**Representative microphotographs of positive surgical margin (PSM) in final histopathologic examination. A**, overview shows densely packed papillary and tubular structures closely approaching the inked surgical margin (arrows). **B**, higher magnification of the marked region in **A** confirms neoplastic tubules reaching the inked surgical margin. Staining: hematoxylin-eosin; scale bar in **A**: 500 μm; scale bar in **B**: 100 μm.

In recent years, organ-sparing surgery for renal tumors in terms of partial nephrectomy or tumor enucleation has replaced radical nephrectomy as the standard procedure for treating locally confined renal cell carcinoma (RCC) [1–8]. This change of therapy is based primarily on findings indicating that organ-sparing surgery offers the potential for better preservation of renal function and a lower risk of cardiovascular sequelae [9–15]. Oncological outcomes appear to be equivalent [3, 16–20], and perioperative morbidity seems to be only minimally higher for nephron-sparing interventions [3, 21, 22]. Just like radical nephrectomy, partial nephrectomy should always aim at complete tumor resection. The width of the normal tissue margin or safety margin around the tumor appears to be of no relevance here [1, 23, 24], but the increased frequency of partial nephrectomies and tumor enucleations has shown that a limited percentage of surgical specimens (between 0 and 7%) have tumor cells in the margin (positive surgical margins, PSM) in the final histopathologic evaluation (Figure 1) [25–27].

However, no prospective and/or randomized study has yet been performed to investigate the prognostic significance of histopathologically positive but intraoperative macroscopically tumor-free surgical margins in predicting the risk of local relapse, metachronous metastases, and tumor-specific survival; only one nonsystematic review has been published thus far [25]. Moreover, most studies had a follow-up of less than five years. This article gives a brief review of currently available data on risk factors for PSM and their potential clinical relevance.

## Review

### Risk factors for positive surgical margins: surgical technique

According to various studies, the incidence of PSM at final pathology is between 0 and 7% for open surgery [28–37], 1 to 4% for laparoscopic interventions [32–34, 38–46], and 4 to 6% [47–49] for robot-assisted surgery. Thus, with appropriate experience and careful patient selection with respect to the optimal surgical approach, PSM rates do not appear to differ significantly between surgical procedures [32–34, 38, 49, 50].

Data from smaller studies suggest that enucleation along the plane of the tumor pseudocapsule may be superior to classic partial nephrectomy with regard to the incidence of PSM. Verze *et al*. [51] retrospectively compared pathological results in cT1 RCC patients after partial nephrectomy (n = 309) and tumor enucleation (n = 226). PSM rates were 6.7% and 1.3% (*P* = 0.01). Multivariate analysis also revealed a nearly five times higher risk of PSM after classic partial nephrectomy (*P* = 0.04). Minervini *et al*. [52] compared pathological results as well as oncological outcomes in RCC patients after partial nephrectomy (n = 982) and tumor enucleation (n = 537) at 16 medical centers. With a median follow-up of approximately four and a half years, the five-year progression-free survival (88.9 versus 91.4%) and tumor-specific survival (93.9 versus 94.3%) did not differ significantly between the two groups, although here too the PSM rate was higher after classic partial nephrectomy (3.4 versus 0.2%).

### Indication

Patients with an imperative indication for nephron-sparing surgery (such as preexisting renal insufficiency, or a functional or anatomical single kidney) have a higher incidence of larger and more unfavorably located tumors than the total patient population. This explains why an imperative indication could be identified as a risk factor for PSM in nearly all studies, at least by univariate analysis. PSM rates of 9 to 28% are described here [5, 53–55]. Using multivariate analysis, Bensalah *et al*. [56] also identified an imperative indication (in addition to tumor location) as an independent risk factor for PSM at final pathology (hazard ratio (HR) 14.3, 95% confidence interval (CI) 1.6 to 21.2; *P* = 0.02).

### Tumor-specific risk factors

According to a study by Kwon *et al*. [29] in 770 patients who underwent open surgery, the PSM rate appears to be unrelated to the histopathological subtype and possibly also the differentiation of RCC. PSM were seen in 33 out of 423 (8%) of all patients with tumors of high malignant potential, and in 24 out of 347 (7%) patients with well-differentiated tumors. In contrast to these findings, Bensalah *et al*. [56] reported a higher incidence of PSM in patients with poorly differentiated carcinomas.

It is still controversial whether tumor size has an impact on the PSM rate. While various research groups were unable to demonstrate a correlation [28, 43], others found higher PSM rates mainly in smaller RCC [57, 58]. Using uni- and multivariate analysis, Yossepowitch *et al*. [57], for example, showed that small tumors are associated more often with PSM but less often with local relapses. It can only be speculated why PSM have been found more often in smaller renal tumors. Possible explanations include the more frequent lack of a pseudocapsule as well as technical inadvertencies during surgical resection or specimen preparation [25]. On the other hand, according to Peycelon *et al*. [5] the PSM rate in very large tumors (more than 7 cm) seems to be rising again. Ani *et al*. [59] have also recently published a study showing a higher PSM rate in patients with larger tumors or a more advanced pathological stage.

It cannot yet be conclusively clarified whether tumor location within the kidney can influence the PSM rate, since none of the published studies included a reproducible nephrometry scoring system. However, available data suggest that PSM is observed more frequently after resection of centrally located tumors [46, 56]. Bensalah *et al*. [56] evaluated 111 patients with and 664 patients without PSM and found positive margins in 26% of all centrally located tumors, but only in 9.1% of all peripherally located tumors (*P* < 0.001).

### Role of intraoperative frozen section analysis

Intraoperative frozen section analysis (FSA) to ensure tumor-free surgical margins is performed frequently and may reduce the rate of PSM, at least in some patient subgroups in laparoscopic surgery (clear cell/papillary subtypes, upper/mid-pole tumors, exophytic/endophytic tumors, pT1a/pT1 tumors, and tumors with histologic differentiation (Fuhrman) grades 1 to 2 and 2 tumors) [60]. However, in the same study, it has also been shown that there is no impact of FSA on patient outcomes except an improved recurrence-free survival in patients with pT1 or exophytic renal cell carcinoma upon laparoscopic surgery, and no effect on tumor recurrence after open surgery. Accordingly, several other studies confirmed that in contrast to individual macroscopic assessment of the tumor bed by the surgeon, intraoperative FSA seems to contribute no decisive information and fails to predict final margin status in cases with a macroscopically tumor-free resection bed [25, 61–65]. Palermo *et al*. proposed quick-staining cytology as an alternative to FSA in a recent publication and showed a good level of agreement with final histologic examination (κ = 0.751; *P* <0.0001) [62].

### Impact of positive surgical margins on local relapses and tumor-specific survival

It has not yet been conclusively clarified whether PSM increase the risk of local relapses after partial nephrectomy, even though the majority of studies suggest that this is probably the case [5, 29, 35, 56, 66]. An overview of published survival analyses is provided in Table 1. Bernhard *et al*. [35] found 26 (3.2%) local relapses in a group of 809 partially nephrectomized patients during a median follow-up of 27 months. In the univariate analysis, the following correlated with local relapse: advanced tumor stage (pT3a), a tumor size greater than 4 cm, imperative indication, bilateral tumors, poor differentiation (Fuhrman histologic differentiation grade of more than 2), and PSM. Bilateral tumors (HR 6.3), tumor size greater than 4 cm (HR 4.6), and especially PSM (HR 11.5) also proved to be independent predictors of ipsilateral relapse. Khalifeh *et al*. [66] even described an 18.4 times higher risk of tumor relapse in 943 patients with a PSM rate of 2.2% after robot-assisted surgery. Kwon *et al*. [29] showed that, in their patient population (n = 770 with 57 (7%) cases of PSM), local relapses only occurred in PSM cases of high-grade malignant tumors.

**Table 1:** **Published hazard ratios (HR) for positive surgical margins in nephron-sparing surgery**

Reference number	Number of cases	HR (recurrence)	HR (OS)	HR (CSS)
[5]	PSM: 22	Not reported	Not reported	Not significant
	NSM: 100			
[68]	PSM: 14	Not reported	Not reported	3.45 (95% CI: 1.79-6.67)
	NSM: 1787			
[67]	PSM: 13	Not reported	Not reported	2.08 (95% CI: 0.84-5.17)
	NSM: 155			
[59]	PSM: 71	Not reported	1.1 (95% CI: 0.66-1.94)	Not significant
	NSM: 587			
[66]	PSM: 21	18.4 (95% CI: 2.27-110.8)	Not reported	Not reported
	NSM: 922			
[57]	PSM: 77	1 (95% CI: 0.23-4.3)	Not reported	Not reported
	NSM: 1313			
[35]	PSM: 12	11.5 (95% CI: 4.66-45.1)	Not reported	Not reported
	NSM: 768			
[56]	PSM: 101	Not significant	Not significant	Not significant
	NSM: 102			

CI, confidence interval; CSS, cancer-specific survival; HR, hazard ratio; NSM, negative surgical margins; OS, overall survival; PSM, positive surgical margins.

Bensalah *et al*. [56] evaluated 111 patients with PSM from various medical centers and established a correlation between PSM and tumor relapse. Time to progression was also shorter in the PSM group (21.4 versus 24.7 months). However, when performing a subsequent matched-pair analysis (n = 101 patients with and n = 102 without PSM), the authors no longer found a significant difference in relapse-free survival (*P* = 0.11) or tumor-specific overall survival (*P* = 0.4). Using multivariate analysis, an imperative indication for partial renal resection (HR 14.3; 95% CI 1.6 to 21.2) and a central tumor location (HR 1.2; 95% CI 1.06 to 1.8) proved to be independent risk factors for tumor relapse, but not PSM at final pathology [56].

In another large study published by Yossepowitch *et al*. [57], 77 out of 1344 (5.7%) patients had PSM; the median follow-up was 3.4 years. In that study too, the risk of local relapse did not differ between patients with or without PSM: five-year rates for freedom from local relapse were 98% and 97%, respectively (*P* = 0.97). Multivariate analysis revealed that, unlike tumor size, PSM was not a risk factor for local relapse (HR 1.0; 95% CI 0.23 to 4.3) or metachronous metastases (HR 1.6; 95% CI 0.6 to 4.1).

A study by Marszalek *et al*. [33] with a median follow-up of 70.7 months showed that, in contrast to tumor size and differentiation, the factor PSM does not predict RCC relapse or overall survival.

In a large study recently published by Ani *et al*. [59], 71 of 664 (10.7%) Canadian patients analyzed retrospectively showed PSM (follow-up of 7.9 years). In that study, the tumor-specific five-year survival rate was 90.9% for patients with PSM and 91.9% for those without PSM (*P* = 0.58). Multivariate analysis also failed to identify PSM status as an independent predictor of cancer-specific survival (HR 1.1; 95% CI 0.66 to 1.94). Thus, microscopic PSM does not appear to significantly influence tumor-specific survival [5, 25, 33, 41, 56, 57, 67]. It has to be noted that in 2002, Frank *et al*. reported a HR of 3.45 (95% CI: 1.79 to 6.67) for cancer-specific survival in patients with PSM; however, one limitation of that study was the small number of PSM cases (n = 14) compared to total cohort size (n = 1801) [68].

## Conclusions

PSM should definitely be avoided even if a certain safety margin is no longer required in nephron-sparing surgery for renal tumors [25, 35]. However, PSM are apparently associated only with an\slightly increased risk of local relapse [33] and are seen especially in RCC that are large [5], poorly differentiated [29, 56], and/or more centrally located [25, 56]. On the other hand, no definite impact on tumor-specific overall survival has as yet been demonstrated [5, 25, 33, 41, 56, 57]. Moreover, a number of studies have already shown that the renal remnant contained a residual tumor in only 0 to 39% of patients with PSM who underwent prophylactic secondary nephrectomy [25, 41, 56, 58, 69, 70]. Consequently, a surveillance strategy rather than a reoperation (repeat resection, completion nephrectomy, or a minimally invasive ablation technique) is now recommended for patients with PSM at final pathology [25, 69].

## Abbreviations

CI: confidence interval
CSS: cancer-specific survival
FSA: frozen section analysis
HR: hazard ratio
NSM: negative surgical margins
OS: overall survival
PSM: positive surgical margins
RCC: renal cell carcinoma
(p)T: (pathological) tumor stage.

## References

[1] Ljungberg B, Cowan NC, Hanbury DC, Hora M, Kuczyk MA, Merseburger AS, Patard JJ, Mulders PF, Sinescu IC (2010). EAU guidelines on renal cell carcinoma: the 2010 update. Eur Urol.

[2] Campbell SC, Novick AC, Belldegrun A, Blute ML, Chow GK, Derweesh IH, Faraday MM, Kaouk JH, Leveillee RJ, Matin SF, Russo P, Uzzo RG (2009). Guideline for management of the clinical T1 renal mass. J Urol.

[3] Minervini A, Serni S, Tuccio A, Siena G, Vittori G, Masieri L, Giancane S, Lanciotti M, Khorrami S, Lapini A, Carini M (2012). Simple enucleation versus radical nephrectomy in the treatment of pT1a and pT1b renal cell carcinoma. Ann Surg Oncol.

[4] Coffin G, Hupertan V, Taksin L, Vaessen C, Chartier-Kastler E, Bitker MO, Roupret M (2011). Impact of elective versus imperative indications on oncologic outcomes after open nephron-sparing surgery for the treatment of sporadic renal cell carcinomas. Ann Surg Oncol.

[5] Peycelon M, Hupertan V, Comperat E, Renard-Penna R, Vaessen C, Conort P, Bitker MO, Chartier-Kastler E, Richard F, Roupret M (2009). Long-term outcomes after nephron sparing surgery for renal cell carcinoma larger than 4 cm. J Urol.

[6] Hansen J, Sun M, Bianchi M, Rink M, Tian Z, Hanna N, Meskawi M, Schmitges J, Shariat SF, Chun FK, Perrotte P, Graefen M, Karakiewicz PI (2012). Assessment of cancer control outcomes in patients with high-risk renal cell carcinoma treated with partial nephrectomy. Urology.

[7] Becker F, Roos FC, Janssen M, Brenner W, Hampel C, Siemer S, Thuroff JW, Stockle M (2011). Short-term functional and oncologic outcomes of nephron-sparing surgery for renal tumours >/=7 cm. Eur Urol.

[8] Hegele A, Skrobek L, Schrader AJ (2014). Update Urooncology: news with clinical relevance from major scientific meetings 2013. Aktuelle Urol.

[9] Huang WC, Levey AS, Serio AM, Snyder M, Vickers AJ, Raj GV, Scardino PT, Russo P (2006). Chronic kidney disease after nephrectomy in patients with renal cortical tumours: a retrospective cohort study. Lancet Oncol.

[10] Klarenbach S, Moore RB, Chapman DW, Dong J, Braam B (2011). Adverse renal outcomes in subjects undergoing nephrectomy for renal tumors: a population-based analysis. Eur Urol.

[11] Scosyrev E, Messing EM, Sylvester R, Campbell S, Van Poppel H (2014). Renal function after nephron-sparing surgery versus radical nephrectomy: results from EORTC randomized trial 30904. Eur Urol.

[12] Huang WC, Elkin EB, Levey AS, Jang TL, Russo P (2009). Partial nephrectomy versus radical nephrectomy in patients with small renal tumors–is there a difference in mortality and cardiovascular outcomes?. J Urol.

[13] Thompson RH, Boorjian SA, Lohse CM, Leibovich BC, Kwon ED, Cheville JC, Blute ML (2008). Radical nephrectomy for pT1a renal masses may be associated with decreased overall survival compared with partial nephrectomy. J Urol.

[14] Weight CJ, Lieser G, Larson BT, Gao T, Lane BR, Campbell SC, Gill IS, Novick AC, Fergany AF (2010). Partial nephrectomy is associated with improved overall survival compared to radical nephrectomy in patients with unanticipated benign renal tumours. Eur Urol.

[15] MacLennan S, Imamura M, Lapitan MC, Omar MI, Lam TB, Hilvano-Cabungcal AM, Royle P, Stewart F, MacLennan G, MacLennan SJ, Canfield SE, McClinton S, Griffiths TR, Ljungberg B, N'Dow J, Ucan Systematic Review Reference Group, E. A. U. Renal Cancer Guideline Panel (2012). Systematic review of oncological outcomes following surgical management of localised renal cancer. Eur Urol.

[16] Van Poppel H, Da Pozzo L, Albrecht W, Matveev V, Bono A, Borkowski A, Colombel M, Klotz L, Skinner E, Keane T, Marreaud S, Collette S, Sylvester R (2011). A prospective, randomised EORTC intergroup phase 3 study comparing the oncologic outcome of elective nephron-sparing surgery and radical nephrectomy for low-stage renal cell carcinoma. Eur Urol.

[17] Minervini A, Di Cristofano C, Lapini A, Marchi M, Lanzi F, Giubilei G, Tosi N, Tuccio A, Mancini M, Della Rocca C, Serni S, Bevilacqua G, Carini M (2009). Histopathologic analysis of peritumoral pseudocapsule and surgical margin status after tumor enucleation for renal cell carcinoma. Eur Urol.

[18] Minervini A, Serni S, Tuccio A, Raspollini MR, Di Cristofano C, Siena G, Vittori G, Saleh O, Lapini A, Carini M (2011). Local recurrence after tumour enucleation for renal cell carcinoma with no ablation of the tumour bed: results of a prospective single-centre study. BJU Int.

[19] Carini M, Minervini A, Masieri L, Lapini A, Serni S (2006). Simple enucleation for the treatment of PT1a renal cell carcinoma: our 20-year experience. Eur Urol.

[20] Steffens S, Junker K, Roos FC, Janssen M, Becker F, Henn D, Wegener G, Siemer S, Hofmann R, Schrader M, Stockle M, Thuroff JW, Hartmann A, Kuczyk MA, Schrader AJ, German Renal Tumor Network (2014). Small renal cell carcinomas - How dangerous are they really? Results of a large multicenter study. Eur J Cancer.

[21] Van Poppel H, Da Pozzo L, Albrecht W, Matveev V, Bono A, Borkowski A, Marechal JM, Klotz L, Skinner E, Keane T, Claessens I, Sylvester R (2007). A prospective randomized EORTC intergroup phase 3 study comparing the complications of elective nephron-sparing surgery and radical nephrectomy for low-stage renal cell carcinoma. Eur Urol.

[22] Schnoeller TJ, De Petriconi R, Hefty R, Jentzmik F, Waalkes S, Zengerling F, Schrader M, Schrader AJ (2011). Partial nephrectomy using porcine small intestinal submucosa. World J Surg Oncol.

[23] Sutherland SE, Resnick MI, Maclennan GT, Goldman HB (2002). Does the size of the surgical margin in partial nephrectomy for renal cell cancer really matter?. J Urol.

[24] Castilla EA, Liou LS, Abrahams NA, Fergany A, Rybicki LA, Myles J, Novick AC (2002). Prognostic importance of resection margin width after nephron-sparing surgery for renal cell carcinoma. Urology.

[25] Marszalek M, Carini M, Chlosta P, Jeschke K, Kirkali Z, Knuchel R, Madersbacher S, Patard JJ, Van Poppel H (2012). Positive surgical margins after nephron-sparing surgery. Eur Urol.

[26] Borghesi M, Brunocilla E, Schiavina R, Martorana G (2013). Positive surgical margins after nephron-sparing surgery for renal cell carcinoma: incidence, clinical impact, and management. Clin Genitourin Cancer.

[27] Masson-Lecomte A, Yates DR, Bensalah K, Vaessen C, de la Taille A, Roumiguie M, Doumerc N, Bruyere F, Soustelle L, Droupy S, Roupret M (2013). Robot-assisted laparoscopic nephron sparing surgery for tumors over 4 cm: operative results and preliminary oncologic outcomes from a multicentre French study. Eur J Surg Oncol.

[28] Patard JJ, Pantuck AJ, Crepel M, Lam JS, Bellec L, Albouy B, Lopes D, Bernhard JC, Guille F, Lacroix B, De La Taille A, Salomon L, Pfister C, Soulie M, Tostain J, Ferriere JM, Abbou CC, Colombel M, Belldegrun AS (2007). Morbidity and clinical outcome of nephron-sparing surgery in relation to tumour size and indication. Eur Urol.

[29] Kwon EO, Carver BS, Snyder ME, Russo P (2007). Impact of positive surgical margins in patients undergoing partial nephrectomy for renal cortical tumours. BJU Int.

[30] Zigeuner R, Quehenberger F, Pummer K, Petritsch P, Hubmer G (2003). Long-term results of nephron-sparing surgery for renal cell carcinoma in 114 patients: risk factors for progressive disease. BJU Int.

[31] Lau WK, Blute ML, Weaver AL, Torres VE, Zincke H (2000). Matched comparison of radical nephrectomy vs nephron-sparing surgery in patients with unilateral renal cell carcinoma and a normal contralateral kidney. Mayo Clinic Proceedings.

[32] Gill IS, Kavoussi LR, Lane BR, Blute ML, Babineau D, Colombo JR, Frank I, Permpongkosol S, Weight CJ, Kaouk JH, Kattan MW, Novick AC (2007). Comparison of 1,800 laparoscopic and open partial nephrectomies for single renal tumors. J Urol.

[33] Marszalek M, Meixl H, Polajnar M, Rauchenwald M, Jeschke K, Madersbacher S (2009). Laparoscopic and open partial nephrectomy: a matched-pair comparison of 200 patients. Eur Urol.

[34] Gong EM, Orvieto MA, Zorn KC, Lucioni A, Steinberg GD, Shalhav AL (2008). Comparison of laparoscopic and open partial nephrectomy in clinical T1a renal tumors. J Endourol.

[35] Bernhard JC, Pantuck AJ, Wallerand H, Crepel M, Ferriere JM, Bellec L, Maurice-Tison S, Robert G, Albouy B, Pasticier G, Soulie M, Lopes D, Lacroix B, Bensalah K, Pfister C, Thuret R, Tostain J, De La Taille A, Salomon L, Abbou C, Colombel M, Belldegrun AS, Patard JJ (2010). Predictive factors for ipsilateral recurrence after nephron-sparing surgery in renal cell carcinoma. Eur Urol.

[36] Duvdevani M, Mor Y, Kastin A, Laufer M, Nadu A, Golomb J, Zilberman D, Nativ O, Ramon J (2006). Renal artery occlusion during nephron-sparing surgery: retrospective review of 301 cases. Urology.

[37] Duvdevani M, Laufer M, Kastin A, Mor Y, Nadu A, Hanani J, Nativ O, Ramon J (2005). Is frozen section analysis in nephron sparing surgery necessary? A clinicopathological study of 301 cases. J Urol.

[38] Gill IS, Matin SF, Desai MM, Kaouk JH, Steinberg A, Mascha E, Thornton J, Sherief MH, Strzempkowski B, Novick AC (2003). Comparative analysis of laparoscopic versus open partial nephrectomy for renal tumors in 200 patients. J Urol.

[39] Lifshitz DA, Shikanov SA, Deklaj T, Katz MH, Zorn KC, Eggener SE, Shalhav AL (2010). Laparoscopic partial nephrectomy: a single-center evolving experience. Urology.

[40] Porpiglia F, Fiori C, Terrone C, Bollito E, Fontana D, Scarpa RM (2005). Assessment of surgical margins in renal cell carcinoma after nephron sparing: a comparative study: laparoscopy vs open surgery. J Urol.

[41] Permpongkosol S, Colombo JR, Gill IS, Kavoussi LR (2006). Positive surgical parenchymal margin after laparoscopic partial nephrectomy for renal cell carcinoma: oncological outcomes. J Urol.

[42] Link RE, Bhayani SB, Allaf ME, Varkarakis I, Inagaki T, Rogers C, Su LM, Jarrett TW, Kavoussi LR (2005). Exploring the learning curve, pathological outcomes and perioperative morbidity of laparoscopic partial nephrectomy performed for renal mass. J Urol.

[43] Porpiglia F, Fiori C, Bertolo R, Scarpa RM (2011). Does tumour size really affect the safety of laparoscopic partial nephrectomy?. BJU Int.

[44] Breda A, Stepanian SV, Liao J, Lam JS, Guazzoni G, Stifelman M, Perry K, Celia A, Breda G, Fornara P, Jackman S, Rosales A, Palou J, Grasso M, Pansadoro V, Disanto V, Porpiglia F, Milani C, Abbou C, Gaston R, Janetschek G, Soomro NA, de la Rosette J, Laguna MP, Schulam PG (2007). Positive margins in laparoscopic partial nephrectomy in 855 cases: a multi-institutional survey from the United States and Europe. J Urol.

[45] Frank I, Colombo JR, Rubinstein M, Desai M, Kaouk J, Gill IS (2006). Laparoscopic partial nephrectomy for centrally located renal tumors. J Urol.

[46] Venkatesh R, Weld K, Ames CD, Figenshau SR, Sundaram CP, Andriole GL, Clayman RV, Landman J (2006). Laparoscopic partial nephrectomy for renal masses: effect of tumor location. Urology.

[47] Scoll BJ, Uzzo RG, Chen DY, Boorjian SA, Kutikov A, Manley BJ, Viterbo R (2010). Robot-assisted partial nephrectomy: a large single-institutional experience. Urology.

[48] Benway BM, Bhayani SB, Rogers CG, Porter JR, Buffi NM, Figenshau RS, Mottrie A (2010). Robot-assisted partial nephrectomy: an international experience. Eur Urol.

[49] Benway BM, Bhayani SB, Rogers CG, Dulabon LM, Patel MN, Lipkin M, Wang AJ, Stifelman MD (2009). Robot assisted partial nephrectomy versus laparoscopic partial nephrectomy for renal tumors: a multi-institutional analysis of perioperative outcomes. J Urol.

[50] Mellon MJ, Lucas SM, Kum JB, Cheng L, Sundaram C (2013). A comparison of pathologic outcomes of matched robotic and open partial nephrectomies. Int Urol Nephrol.

[51] Verze P, Fusco F, Minervini A, Antonelli A, Bianchi G, Bocciardi A, Cosciani Cunico S, Ficarra V, Fiori C, Giancane S, Longo N, Martorana G, Novara G, Porpiglia F, Rocco F, Rovereto B, Schiavina R, Serni S, Simeone C, Volpe A, Carini M (2013). Simple enucleation versus standard partial nephrectomy for clinical T1 renal tumors: Intraoperative, early post-operative and pathological outcomes from a prospective multicenter comparative study (RECORd Project). Eur Urol Suppl.

[52] Minervini A, Ficarra V, Rocco F, Antonelli A, Bertini R, Carmignani G, Cosciani Cunico S, Fontana D, Longo N, Martorana G, Mirone V, Morgia G, Novara G, Roscigno M, Schiavina R, Serni S, Simeone C, Simonato A, Siracusano S, Volpe A, Zattoni F, Zucchi A, Carini M (2011). Simple enucleation is equivalent to traditional partial nephrectomy for renal cell carcinoma: results of a nonrandomized, retrospective, comparative study. J Urol.

[53] Saranchuk JW, Touijer AK, Hakimian P, Snyder ME, Russo P (2004). Partial nephrectomy for patients with a solitary kidney: the Memorial Sloan-Kettering experience. BJU Int.

[54] Lee DJ, Hruby G, Benson MC, McKiernan JM (2011). Renal function and oncologic outcomes in nephron sparing surgery for renal masses in solitary kidneys. World J Urol.

[55] Wszolek MF, Kenney PA, Lee Y, Libertino JA (2011). Comparison of hilar clamping and non-hilar clamping partial nephrectomy for tumours involving a solitary kidney. BJU Int.

[56] Bensalah K, Pantuck AJ, Rioux-Leclercq N, Thuret R, Montorsi F, Karakiewicz PI, Mottet N, Zini L, Bertini R, Salomon L, Villers A, Soulie M, Bellec L, Rischmann P, De la Taille A, Avakian R, Crepel M, Ferriere JM, Bernhard JC, Dujardin T, Pouliot F, Rigaud J, Pfister C, Albouy B, Guy L, Joniau S, van Poppel H, Lebret T, Culty T, Saint F (2010). Positive surgical margin appears to have negligible impact on survival of renal cell carcinomas treated by nephron-sparing surgery. Eur Urol.

[57] Yossepowitch O, Thompson RH, Leibovich BC, Eggener SE, Pettus JA, Kwon ED, Herr HW, Blute ML, Russo P (2008). Positive surgical margins at partial nephrectomy: predictors and oncological outcomes. J Urol.

[58] Raz O, Mendlovic S, Shilo Y, Leibovici D, Sandbank J, Lindner A, Zisman A (2010). Positive surgical margins with renal cell carcinoma have a limited influence on long-term oncological outcomes of nephron sparing surgery. Urology.

[59] Ani I, Finelli A, Alibhai SM, Timilshina N, Fleshner N, Abouassaly R (2013). Prevalence and impact on survival of positive surgical margins in partial nephrectomy for renal cell carcinoma: a population-based study. BJU Int.

[60] Venigalla S, Wu G, Miyamoto H (2013). The impact of frozen section analysis during partial nephrectomy on surgical margin status and tumor recurrence: a clinicopathologic study of 433 cases. Clin Genitourin Cancer.

[61] Timsit MO, Bazin JP, Thiounn N, Fontaine E, Chretien Y, Dufour B, Mejean A (2006). Prospective study of safety margins in partial nephrectomy: intraoperative assessment and contribution of frozen sections [abstract 638]. Eur Urol Suppl.

[62] Palermo SM, Dechet C, Trenti E, Mian C, Lodde M, Comploj E, Mazzoleni G, Hanspeter E, Ambrosini Spaltro A, Mayr R, Pycha A (2013). Cytology as an alternative to frozen section at the time of nephron-sparing surgery to evaluate surgical margin status. Urology.

[63] Sterious SN, Simhan J, Smaldone MC, Tsai KJ, Canter D, Wameedh E, Li T, Helstrom J, Viterbo R, Chen DY, Greenberg RE, Kutikov A, Al-Saleem T (2013). Is there a benefit to frozen section analysis at the time of partial nephrectomy?. Can J Urol.

[64] Hillyer SP, Yakoubi R, Autorino R, Isac W, Miocinovic R, Laydner H, Khalifeh A, Stein RJ, Haber GP, Kaouk JH (2013). Utility of intraoperative frozen section during robot-assisted partial nephrectomy: a single institution experience. J Endourol.

[65] Hagemann IS, Lewis JS (2009). A retrospective comparison of 2 methods of intraoperative margin evaluation during partial nephrectomy. J Urol.

[66] Khalifeh A, Kaouk JH, Bhayani S, Rogers C, Stifelman M, Tanagho YS, Kumar R, Gorin MA, Sivarajan G, Samarasekera D, Allaf ME (2013). Positive surgical margins in robot-assisted partial nephrectomy: a multi-institutional analysis of oncologic outcomes (leave no tumor behind). J Urol.

[67] Haferkamp A, Kurosch M, Pritsch M, Hatiboglu G, Macher-Goeppinger S, Pfitzenmaier J, Pahernik S, Wagener N, Hohenfellner M (2010). Prognostic factors influencing long-term survival of patients undergoing nephron-sparing surgery for nonmetastatic renal-cell carcinoma (RCC) with imperative indications. Ann Surg Oncol.

[68] Frank I, Blute ML, Cheville JC, Lohse CM, Weaver AL, Zincke H (2002). An outcome prediction model for patients with clear cell renal cell carcinoma treated with radical nephrectomy based on tumor stage, size, grade and necrosis: the sign score. J Urol.

[69] Sundaram V, Figenshau RS, Roytman TM, Kibel AS, Grubb RL, Bullock A, Benway BM, Bhayani SB (2011). Positive margin during partial nephrectomy: does cancer remain in the renal remnant?. Urology.

[70] Ray ER, Turney BW, Singh R, Chandra A, Cranston DW, O'Brien TS (2006). Open partial nephrectomy: outcomes from two UK centres. BJU Int.

